# Usage and parental knowledge of antibiotics in children undergoing (adeno) tonsillectomy in northern Tanzania

**DOI:** 10.11604/pamj.2023.46.59.41190

**Published:** 2023-10-17

**Authors:** Denis Robert Katundu, Gerjon Hannink, Jesca Godlisten Lyimo, Maroeska Rovers, Niels van Heerbeek

**Affiliations:** 1 Department of Otolaryngology, Kilimanjaro Christian Medical Centre, Kilimanjaro, Tanzania,; 2 Department of Otolaryngology, Kilimanjaro Christian Medical University College, Kilimanjaro, Tanzania,; 3 Department of Otolaryngology, Head and Neck Surgery, Radboudumc, Nijmegen, The Netherlands,; 4 Department of Medical Imaging, Radboudumc, Nijmegen, The Netherlands

**Keywords:** Upper respiratory tract infections, tonsilitis, antibiotics, adenotonsillectomy

## Abstract

**Introduction:**

Antimicrobial Resistance (AMR) is a growing concern globally, mostly being contributed by a limited understanding of antibiotic utilization as a result of inappropriate acquisition and prescription. Parental awareness is essential in optimizing their usage and preserving the effectiveness of these crucial medications. The current study investigates the usage and parental knowledge of antibiotics in children undergoing (adeno) tonsillectomy ((A)TE) in Northern Tanzania.

**Methods:**

a cross-sectional survey was conducted among parents/caregivers of children who underwent (A)TE in Northern Tanzania. A modified and well-structured questionnaire, which was adapted from a World Health Organization (WHO) questionnaire and used to assess the parents´ knowledge of antibiotics and antibiotic use.

**Results:**

the study included 157 participants. About 54% of the children under the age of 5 years. As of 88% of children had already received antibiotics prior to surgery, 92% of the used antibiotics were prescribed by a clinician, and 5% of parents to used leftovers antibiotics for their children. While 88% of the parents reported adhering to prescriptions, 8% of reported buying the same antibiotic (as prescribed before) without consulting a clinician again when their children are sick.

**Conclusion:**

the use of antibiotics, including broad-spectrum antibiotics, was found to be high in our study group. Parents demonstrate a relatively good understanding of antibiotic usage. It is plausible to speculate that a higher prevalence of non-insured, unemployed, and less educated parents may lead to an increased incidence of misuse and misinterpretation of antibiotics.

## Introduction

Antimicrobial resistance (AMR) is a growing problem worldwide due to improper antimicrobial prescription and use, particularly in low- and middle-income countries (LMIC) [1-4]. Children younger than 5 years account for up to 80% of those treated with antibiotics for upper respiratory tract infections (URTI), despite evidence showing that a significant number of children with URTI do not need antimicrobial agents [5,6]. In LMIC, there is still an assumption that antibiotics are effective for treating every child with URTI [7-9]. This could be due to poor understanding of the effects of antibiotics, and poor antimicrobial stewardship [6,10,11]. Parental knowledge of antibiotics and their use in the treatment of URTI and pharyngotonsillitis in children is precarious and undetermined in Tanzania, and data regarding this topic are scarce in both Tanzania and other LMIC.

Infections of adenoids and tonsils, alone or as part of upper respiratory infection, are the main reason for children seeking medical attention in most LMICs. It is estimated that nearly all children receive a course of antibiotics, regardless of the clinical presentation or, if done, laboratory microbiological results [1,6,12]. The majority of caretakers and parents tend to present their children for medical attention due to sore throat and sleep apnoea [13]. However, acute tonsillopharyngitis, being the most common cause of sore throat, is often viral, and a bacterial etiology is only responsible for a small percentage of cases [14,15]. Of those bacterial pathogens, group A β-haemolytic streptococcus (GABHS) is a common cause of tonsillopharyngitis. In children, the incidence ranges between 15-30% [14,16,17]. Studies have shown that the rate of prescribing antibiotics for GABHS is unnecessarily high [18-20].

Although it is sometimes difficult to differentiate between viral and bacterial etiologies of tonsillopharyngitis, appropriate history taking together with physical and laboratory findings might be helpful [3,21,22]. Clinical presentation of adenotonsillitis amongst other respiratory diseases has provided a mechanism for antimicrobial misprescription in many parts of the developing world.

Several guidelines, such as those by the UK´s National Institute for Health and Care Excellence (NICE), Dutch, German, and American Centers for Disease Control and Prevention (CDC), recommend against the unnecessary use of antimicrobial agents in pharyngotonsillitis as well as other upper respiratory tract infections [18,20,23-25]. These guidelines recommend antibiotics only in cases of severe infection or strongly suspected GABHS infection [20,23-25]. However, this is contrary to the Tanzanian National Standards Treatment Guideline (STG) that advocates antibiotics regardless of clinical and laboratory arguments [14,24-26]. It is estimated that there is a major underreporting of antibiotic misuse both from healthcare providers and consumers. Following the introduction of Accredited Drug Dispensing Outlets (ADDO) 20 years ago, Tanzanians have increased and easy access to antibiotics. ADDO are certified stores and have aided in increasing access to essential drugs in rural and peri-urban areas. Due to their profit making practice, poor adherence to policy, and lack of timely inspection and supervision ADDOs have been a major source of unprescribed antibiotics, and as such they have been contributing to AMR [1,4,5,8,18].

A hospital-based survey was conducted to study the usage of antibiotics and parental knowledge on antibiotic use among children with recurrent chronic tonsillitis and/or tonsillar hypertrophy who are scheduled for (adeno) tonsillectomy ((A)TE) at a tertiary-level academic hospital.

## Methods

**Study design:** a cross-sectional survey was conducted.

**Setting:** this study was conducted at a tertiary academic hospital (Kilimanjaro Christian Medical Centre (KCMC)) that serves approximately 6 million people in Northern Tanzania, between March and October 2022.

**Participants:** parents who brought their children to the Ear, Nose and Throat (ENT) outpatient clinic to see one of the authors (DK and/or JL) and who met the study inclusion criteria.

**Variables:** demographic data were collected from both the child and parents. The number of throat infections/URTI as well as the use of antibiotics during the 12 months prior to visiting the hospital were collected. The number of antibiotic courses taken, type of antibiotic(s), and how the antibiotic(s) were obtained was registered.

**Data sources/measurement:** a modified and well-structured questionnaire, which was adapted from a WHO questionnaire that was used in a multi-country awareness survey, was used to assess the parents´ knowledge of antibiotics and antibiotic use [6,27]. Parents who gave written consent were asked to complete the printed study questionnaire with 20 questions (Annex 1) [28].

**Bias:** our study was based on self-reported data, that may be subject to courtesy and recall biases.

**Study size:** a total of 173 children who attended our outpatient clinic were enrolled in this study of which, 157 were included after meeting inclusion criteria and consented to participate.

**Inclusion and exclusion criteria:** ages between 1 and 18 years scheduled for (A)TE due to (obstructive) sleep-disordered breathing as a result of adenotonsillar hypertrophy and/or recurrent chronic tonsillitis without known immunodeficiency syndrome(s) were included.

**Quantitative variables:** descriptive statistics were used to summarize the data. Continuous variables were expressed as means and standard deviations, while categorical variables were expressed as frequencies and percentages.

**Statistical methods:** statistical analyses were performed using R (version 4.2.0; R Foundation for Statistical Computing, Vienna, Austria).

**Ethical consideration:** ethical approval secured from hospital's ethical committee. This study was reported according to the Strengthening the Reporting of Observational Studies in Epidemiology (STROBE) Statement.

## Results

**Participants:** one hundred and fifty-seven parents consented to participate in the survey.

**Descriptive data:** Table 1 provides the demographic characteristics of the children in the study. More than half (54%) of the children were below the age of 5 years.

**Table 1:** demographic characteristics of study participants, recruited from the Ear Nose and Throat (ENT) Department of Kilimanjaro Christian Medical Centre (Tanzania), from March to October 2022 (N=157)

Age (group)	N (%)
1-5	85 (54.1)
6-11	65 (41.3)
12-18	7 (4.3)
**Sex**	
Female	79 (50.3)
Male	78 (49.7)
Weight (kg) (mean (SD))	20.18 (8.27)
**Level of education**	
Pre-school	122 (77.7)
Primary	31 (19.7)
Secondary	4 (2.5)
**Health/medical insurance status**	
Yes	141 (89.8)
No	16 (10.2)
**Marital status of parents (%)**	
Married	141 (89.8)
Divorced	2 (1.3)
Single parenthood	14 (8.9)
**Highest level of education father**	
University	62 (39.5)
College	49 (31.2)
Secondary	36 (22.9)
Primary	6 (3.8)
Pre-school	2 (1.3)
No-formal education	2 (1.3)
**Employment status, father**	
Employed by parastatal/private/government organization	104 (66.2)
Self-employed	46 (29.3)
Unemployed	7 (4.5)
**Highest level of education, mother**	
University	31 (19.7)
College	63 (40.1)
Secondary	55 (35.0)
Primary	6 (3.8)
Pre-school	1 (0.6)
No-formal education	1 (0.6)
**Employment status mother**	
Employed by parastatal/private/government	82 (52.2)
Self-employed	62 (39.5)
Unemployed	13 (8.3)
**Scheduled surgery**	
Tonsillectomy	20 (12.8)
Adenotonsillectomy	137 (87.2)
**BMI category by WHO (%)**	
Underweight	49 (31.2)
Normal weight	43 (27.4)
Pre-obesity	37 (23.6)
Obesity class I	25 (15.9)
Obesity class II	3 (1.9)

BMI: body mass index; WHO: World Health Organization

**Outcome data:** about 90% of children reported to have health insurance coverage, with the majority covered by the national insurance scheme National Health Insurance Fund (NHIF). About 40% of fathers and 30% of mothers reported to have attended college, and more than 50% of both parents were employed in the formal sector (i.e. employed by parastatal/private/government organizations) while 29% and 39% of fathers and mothers were in informal employments (i.e. self-employed as entrepreneur/driver/tailor/ crafts (wo) man/seller), respectively.

**Main results:** over a twelve-month period, children reported a median number of 4.00 (IQR 3.00 - 5.00) episodes of throat/upper respiratory tract infections prior to visiting the hospital. In total 88% of the children used at least one antibiotic course during that year. A total of 232 courses were used, resulting in a median number of 1.00 (IQR 1.00 - 2.00) antibiotics per child over the last twelve months (Table 2). About 43% of the children who participated were prescribed at least two doses of antibiotics before visiting an ENT practitioner, some were prescribed even broad-spectrum antibiotics. Amoxicillin/clavulanic acid was the most commonly used antibiotic, followed by azithromycin. Other antibiotics taken are described in Table 3. Although most antibiotics were prescribed by a clinician (92%), about 5% were leftovers from a previous prescription(s), while others were either borrowed, shared from a friend/colleague/family member, or bought over the counter without proper prescription. Most parents (88%) ensured that their children finished the prescribed antibiotic. However, about 8% believed it was okay to obtain the same course of antibiotics without visiting the clinician if their child showed similar signs and symptoms in the future.

**Table 2:** antibiotic prescribing practices and parental knowledge of antibiotic use among 157 children with throat and upper respiratory tract infections, recruited from the Ear Nose and Throat (ENT) Department of Kilimanjaro Christian Medical Centre (Tanzania), from March to October 2022 (N=157)

Characteristics	n (%)
**Antibiotics prescribed?**	
Yes	139 (88.5)
No	18 (11.5)
**Number of throat infections/URTI in the previous year per child**	
0	4 (2.5)
1	4 (2.5)
2	17 (10.8)
3	38 (24.2)
4	28 (17.8)
5	30 (19.1)
6	19 (12.1)
7	10 (6.4)
8	2 (1.3)
9	0 (0)
10	1 (0.6)
11	4 (2.5)
**Number of antibiotic courses per child**	
0	17 (10.8)
1	71 (45.2)
2	49 (31.2)
3	17 (10.8)
4	3 (1.9)
**How were antibiotics obtained?**	
Prescribed by doctor/clinician	145 (92.4)
Used the previous “leftover”	8 (5.1)
Bought over the counter	3 (1.9)
Borrowed/shared from friend/colleague/family member	1 (0.6)
**When to stop antibiotics?**	
When you finish the dose as directed	139 (88.5)
When you feel better	18 (11.5)
**Is it okay to use antibiotics given to someone else?**	
No	157 (96.8)
Yes	5 (3.2)
**Is it okay to use the same antibiotic as before without consulting a clinician again?**	
No	144 (91.7)
Yes	13 (8.3)

URTI: upper respiratory tract infections; SD: standard deviation

**Table 3:** antibiotic use among 157 children with throat and upper respiratory tract infections during the 12 months preceding (adeno) tonsillectomy recruited from the Ear Nose and Throat (ENT) Department of Kilimanjaro Christian Medical Centre (Tanzania), from March to October 2022 (N=157)

Antibiotic	n (%)
Amoxicillin/clavulanic acid	86 (37.8)
Azithromycin	60 (26.4)
Ampicillin	31 (.3)
Ampicillin/cloxacillin	21 (9.2)
Amoxicillin/flucloxacillin	17 (7.4)
Amoxicillin	14 (6.1)
Ceftriaxone	13 (5.7)
Penicillin V	4 (1.7)
Cephalexin	2 (0.8)
Penicillin	2 (0.8)
Do not remember	5 (2.2)
Total	227 (100)

## Discussion

The aim of this study was to investigate the usage of antibiotics and the level of knowledge regarding antibiotic use among parents of children who were scheduled to undergo (A)TE. The use of antibiotics, including broad-spectrum antibiotics, was found to be high in our study group. The majority of study participants were children of employed parents or those under the voluntary national health insurance fund, which costs approximately 18 US dollars per year [29,30]. These participants had access to medical and surgical services at this tertiary academic hospital at no additional cost. However, other medical centers do not accept these insurances for A(TE) purposes as they are underpaid by the insurer. In such cases, the insured has to either subside the difference or pay the whole amount [30-32]. Reports show that only 32% of Tanzanian people have health insurance [29,31,33,34]. Therefore, the group of children without insurance is underrepresented in this survey. Therefore, our findings should be interpreted with caution.

Reports show that about 28.6% and 7.8% of the general population enroll in secondary school and tertiary education respectively [35]. In our current study, most parents had finished tertiary education. Most of those parents were aware that antibiotics must be prescribed by doctors and that the full course of medication should be finished. This knowledge may probably be attributed to their level of education, hence influencing their knowledge towards antibiotic use. Their relatively high level of education and employment also may explain the high percentage of health insurance [15,30,36]. Nevertheless, this study shows that antibiotics are still being over-prescribed in children in Northern Tanzania for conditions such as sore throat and/or URTI, including the prescription of even broader antimicrobials, such as third line cephalosporins [9,37].

There are several reasons why improper practices regarding antibiotic prescription and use continue to occur. Among the reasons, are the insufficient evidence-based national treatment guidelines that advocates the use of antibiotic without thorough clinical and microbiological bases [9,26,38]. Other East African countries guidelines advocate a more strict policy towards the use of antibiotics for URTI and pharyngotonsillitis [39-42]. Reports show that even the availability of effective guidelines does not seem to be the single factor to significantly affects trends towards proper antibiotic prescription. That is why other factors should be considered in taking measures on antimicrobial stewardship [43]. Many practitioners in Tanzania adhere well to STG that emphasizes on prescription of antibiotic in acute and chronic tonsilitis and URTI [5,20,37]. We strongly believe that the STG is outdated and lacks clarity towards antibiotic prescription in the treatment of throat and/or URTI. Poor antimicrobial stewardship strategies which have enabled easy prescription, access, and dispensing of antibiotics, also contribute to misprescription. The use of leftover and sharing antibiotics is also practiced by some parents. This may be due to limited knowledge of antimicrobial use especially in rural areas, being attributed to poor health-seeking behavior among people with financial constraints and lower levels of education. Understanding the cost implication and hazards of AMR particularly in children, strict measures should be taken by the government of Tanzania through its regulatory authorities [4,36].

It is worth noting that our study has limitations. Our study was based on self-reported data, that may be subject to courtesy and recall biases. Additionally, the survey was performed at a tertiary referral academic hospital that is affordable for mostly insured patients and only a few patients paying out of pocket, which may limit the generalizability of the findings to the rest of the Tanzanian population. Nonetheless, having a properly updated national treatment guideline will aid in antibiotic stewardship. Further education on proper antibiotic use will also greatly contribute to combating AMR.

**Funding and role of the funding source:** all costs are mainly covered by a grant from the Radboudumc Revolving Research Fund (R3Fund). Funder of the study had no role in trial design, data collection, data analysis, data interpretation, or writing of the report.

## Conclusion

The use of antibiotics, including broad-spectrum antibiotics, was found to be high in our study group. Although parents demonstrate a relatively good understanding of antibiotic usage, it is plausible to speculate that a higher prevalence of non-insured, unemployed, and less educated parents may lead to an increased incidence of misuse and misinterpretation of antibiotics. Considering the potential influence of socio-economic factors, educational programs should be directed towards this specific group to enhance understanding of appropriate antibiotic use, without leaving behind prescribing clinicians and dispensing personnel. Updating STG in Tanzania and adhering to evidence-based antibiotic prescribing principles offer a promising strategy for effectively managing URTI and tonsillitis, while simultaneously curbing unnecessary antibiotic prescriptions. A comprehensive approach involving healthcare professionals, policymakers, and the community is crucial in promoting judicious antibiotic use and safeguarding public health.

### What is known about this topic

- *Upper respiratory tract infections (URTIs) primarily involve viral infections affecting the nose, throat, sinuses, and upper airways;*
- *Antibiotics are frequently overprescribed for URTIs and pharyngotonsillitis, even when viral causes are likely;*
- *Antibiotic overuse/misuse can lead to antibiotic resistance, a global health concern; proper diagnosis of bacterial infections before prescribing antibiotics is crucial*.

### What this study adds

- *High use of antibiotics, including broad-spectrum antibiotics, in URTI and tonsillitis treatment among children;*
- *Parents demonstrate good antibiotic understanding, but higher misuse is associated with non-insured, unemployed, and less educated parents;*
- *Educational programs targeting socio-economically disadvantaged groups can improve appropriate antibiotic use; updating STG and adhering to evidence-based prescribing principles are promising strategies*.

## Annex

**Annex 1**: questionnaire for study assessing antibiotic use in pediatric pre (a)te patients (PDF - 99 Kb)
